# Septic Shock Due to Liver Abscess Complicated by Recurrent Heart Failure Exacerbation in a Patient Diagnosed With Heart Failure With Preserved Ejection Fraction (HFpEF)

**DOI:** 10.7759/cureus.109150

**Published:** 2026-05-18

**Authors:** Haruna Mitani, Akiyo Horiguchi, Taichi Fujimori, Ryuichi Ohta, Chiaki Sano

**Affiliations:** 1 Medicine, Shimane University Faculty of Medicine, Izumo, JPN; 2 Community Care, Unnan City Hospital, Unnan, JPN; 3 Community Medicine Management, Shimane University Faculty of Medicine, Izumo, JPN

**Keywords:** abscess drainage, elderly patient, fluid management, hemodynamic instability, hfpef, liver abscess, septic shock, source control

## Abstract

Liver abscess is a severe infection that can progress to septic shock. Although early fluid resuscitation is recommended in septic shock, patients with heart failure, particularly those with heart failure with preserved ejection fraction (HFpEF), are prone to fluid overload, making volume management challenging. In addition, hemodynamic instability may occur even after source control. We report the case of a 91-year-old man with a history of HFpEF who was transported to our hospital with fever and difficulty ambulating. He was diagnosed with septic shock secondary to a liver abscess caused by *Escherichia coli*. Initial resuscitation was performed with antibiotics, vasopressors, and approximately 6.5 L of intravenous fluid, followed by percutaneous drainage. Although his hemodynamic status temporarily improved, shock recurred after the drainage procedure. During hospitalization, he experienced multiple episodes of heart failure exacerbation associated with fluid shifts, requiring careful fluid management, including diuretics and respiratory support. His general condition subsequently improved, and he was successfully weaned from intensive care. This case suggests the importance of balancing adequate initial resuscitation with avoidance of fluid overload in septic shock complicated by HFpEF, and the potential for transient hemodynamic deterioration following source control. Individualized fluid management and close periprocedural monitoring are essential in such patients.

## Introduction

Liver abscess is a disease characterized by the formation of a purulent collection within the hepatic parenchyma due to bacterial infection and is more commonly observed in elderly individuals and patients with diabetes mellitus [1-3]. The most common causative organisms include *Escherichia coli* and *Klebsiella pneumoniae*, and the primary routes of infection are biliary tract infection and portal venous spread [1-3]. Clinical manifestations are often nonspecific, including fever, abdominal pain, and malaise, but severe cases may progress to sepsis or septic shock [1].

Imaging studies are essential for diagnosis, and treatment consists of antimicrobial therapy in combination with drainage as a key component of source control [1]. Although aggressive fluid resuscitation is recommended in the initial management of septic shock [4], elderly patients and those with heart failure are prone to fluid overload due to impaired diastolic function and reduced vascular compliance [5,6], which can complicate fluid management. Furthermore, it is recognized that transient worsening of the inflammatory response after source control may lead to recurrence of shock.

In this report, we describe a case of septic shock secondary to a liver abscess in which repeated exacerbations of heart failure occurred in the context of underlying cardiac dysfunction, and shock recurred after drainage. This case highlights the importance of careful fluid management and periprocedural hemodynamic monitoring in the management of sepsis.

## Case presentation

A 91-year-old man (height 168 cm, weight 58.0 kg), who was independent in activities of daily living and living with his daughter, was brought to our hospital by ambulance due to fever and difficulty with ambulation. He had developed a fever two days before admission and experienced vomiting the day before the presentation. Until three days before admission, he had been eating normally; however, his oral intake had decreased on the day before admission. On the day of presentation, his daughter found him unable to move upon returning home and called emergency medical services.

His past medical history included right pneumothorax in his 90s, constipation, diabetes mellitus, hypertension, acute myocardial infarction in his 50s (treated with stent placement), heart failure, tuberculosis in his 60s, pulmonary emphysema, cholecystitis eight years before (status post cholecystectomy), and ischemic colitis. His medications included aspirin 100 mg once daily, lansoprazole orally disintegrating tablet 15 mg once daily, sitagliptin 25 mg once daily, allopurinol 50 mg once daily, atorvastatin 20 mg once daily, sacubitril/valsartan 200 mg once daily, magnesium oxide 660 mg once daily, and suvorexant 15 mg once daily. He had a smoking history of 20 cigarettes per day until age 60, consumed alcohol occasionally, and had no known drug or food allergies.

Physical examination

On arrival, he was fully alert. His body temperature was 37.8°C, blood pressure was 74/44 mmHg (which improved to 94/49 mmHg after administration of 500 mL of intravenous fluids), heart rate was 85 beats/minute, respiratory rate was 20 breaths/minute, and oxygen saturation was 95% on room air. His general condition was relatively preserved. There was no conjunctival pallor or scleral icterus. No neck stiffness was noted, and jolt accentuation was negative. The abdomen was flat and soft, without tenderness, liver percussion pain, or costovertebral angle tenderness. No surgical scars were noted. The extremities were cool, but not clammy, and radial pulses were palpable. No mottling was observed. There were no focal neurological deficits or skin rashes.

Transthoracic echocardiography demonstrated preserved left ventricular systolic function and a collapsed inferior vena cava. Abdominal ultrasonography revealed no ascites. No gallbladder enlargement was observed. Bladder volume was low, and urinary retention was not present.

Investigations and diagnosis

Laboratory findings on admission showed marked inflammatory response, with a white blood cell count of 13.3 × 10³/μL and neutrophils at 93.4%. C-reactive protein was elevated at 11.66 mg/dL, and lactate was 6.0 mmol/L. Platelet count was decreased to 8.8 × 10⁴/μL. Renal function was impaired, with a creatinine level of 2.41 mg/dL, indicating acute kidney injury. Mild elevations in hepatobiliary enzymes were noted (aspartate aminotransferase 178 U/L, total bilirubin 1.4 mg/dL). Arterial blood gas analysis showed a P/F ratio of 356 (Table 1).

**Table 1:** Initial laboratory data of the patient (HCU Day 1) Initial laboratory findings showed leukocytosis with neutrophilia, thrombocytopenia, elevated inflammatory markers, hyperlactatemia, hepatobiliary enzyme elevation, and acute kidney injury, consistent with septic shock CRP: C-reactive protein; HCU: high care unit

Parameter	Level	Reference range
White blood cells	13.30 × 10^3^/μL	3.5-9.8 × 10^3^/μL
Neutrophils	93.4%	44.0%-72.0%
Lymphocytes	2.6%	18.0%-59.0%
Hemoglobin	11.2 g/dL	13.5-17.6 g/dL
Hematocrit	34.7%	36%-48%
Mean corpuscular volume	101.6 fL	82-101 fL
Platelets	8.8 × 10^4^/μL	13.0-36.9 × 10^4^/μL
Total protein	6.1 g/dL	6.6-8.1 g/dL
Albumin	2.9 g/dL	3.9-4.9 g/dL
Total bilirubin	1.4 mg/dL	0.2-1.2 mg/dL
Aspartate aminotransferase	178 IU/L	8-38 IU/L
Alanine aminotransferase	44 IU/L	4-44 IU/L
Lactate dehydrogenase	364 U/L	106-211 U/L
Blood urea nitrogen	39.3 mg/dL	8.0-20.0 mg/dL
Creatinine	2.41 mg/dL	0.40-1.10 mg/dL
Serum Na	133 mEq/L	135-147 mEq/L
Serum K	4.3 mEq/L	3.3-4.8 mEq/L
Serum Cl	97 mEq/L	98-108 mEq/L
CRP	11.66 mg/dL	<0.30 mg/dL
PaO_2_/FIO_2_	356	-
Lactate	6.0 mmol/L	0.5-1.6 mmol/L

Blood cultures grew *E. coli*. Contrast-enhanced computed tomography (CT) revealed a hepatic abscess with bile duct dilatation (Figure 1). A corresponding low-attenuation lesion was identified on noncontrast CT obtained two months before admission (Figure 2), consistent with a preexisting hepatic cyst. *E. coli* was also isolated from the aspirated fluid of the hepatic lesion. Based on these findings, the patient was diagnosed with septic shock secondary to *E. coli* bacteremia originating from a liver abscess. The lesion was considered likely to represent an infected preexisting hepatic cyst that had progressed to an abscess.

**Figure 1:**
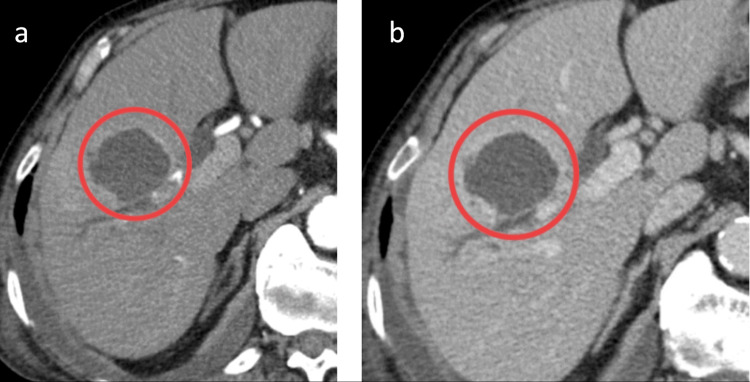
Contrast-enhanced abdominal CT demonstrating a liver abscess (HCU Day 3) Contrast-enhanced axial CT images of the abdomen on HCU Day 3. (a) Arterial phase image showing a well-defined low-attenuation hepatic abscess with peripheral rim enhancement (red circle). (b) Portal venous phase image demonstrating the same hepatic abscess with persistent rim enhancement (red circle) CT: computed tomography; HCU: high care unit

**Figure 2:**
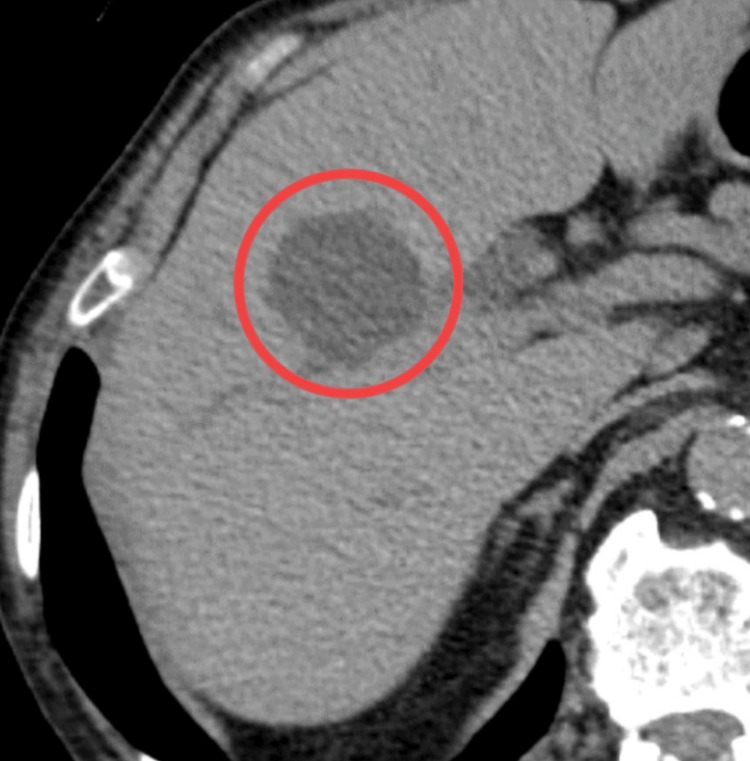
Noncontrast axial abdominal CT obtained two months before admission A well-defined low-attenuation lesion is observed in the liver (red circle), consistent with a hepatic cyst. This lesion corresponds to the site of the liver abscess identified on contrast-enhanced CT (as shown in Figure 1) CT: computed tomography

Clinical course

During hospitalization, the platelet count decreased from 8.8 × 10⁴/μL on admission (high care unit (HCU), an intermediate care unit equivalent to a step-down unit, Day 1) to 6.4 × 10⁴/μL on HCU Day 3, followed by gradual recovery to 9.9 × 10⁴/μL by HCU Day 7. Total bilirubin increased from 1.4 mg/dL at admission to 1.7 mg/dL on HCU Day 2, then improved and stabilized at 0.6-0.7 mg/dL. Renal function improved from a creatinine level of 2.41 mg/dL on admission to 0.74 mg/dL by HCU Day 7. Lactate levels decreased from 6.0 mmol/L on admission to 1.2 mmol/L by HCU Day 4 and remained within the normal range thereafter. The P/F ratio declined from 356 on admission to 203 on HCU Day 3 and further to 181, followed by recovery to 287 by HCU Day 8.

Treatment and outcome

For septic shock, tazobactam/piperacillin (TAZ/PIPC) was initiated on HCU Day 1. On the same day, approximately 6.5 L of intravenous fluids were administered along with vasopressor support, requiring norepinephrine up to 0.05 μg/kg/minute and vasopressin at 1 U/hour (0.017 U/minute). As acute cholangitis was initially considered, contrast-enhanced CT on HCU Day 3 clarified hepatic abscess as the primary source of infection, leading to the selection of percutaneous drainage rather than endoscopic retrograde cholangiopancreatography for definitive source control. Culture of the aspirated fluid yielded *E. coli* (3+), consistent with the blood culture findings.

Although the septic shock initially improved, the patient developed chills and hemodynamic deterioration after drainage, with recurrence of septic shock accompanied by mottling, necessitating escalation of norepinephrine to a maximum of 0.10 μg/kg/minute.

Given his history of heart failure with preserved ejection fraction (HFpEF), he experienced multiple episodes of heart failure exacerbation due to fluid loading and increased afterload from vasopressors. The first exacerbation occurred on HCU Day 2 and improved with noninvasive positive pressure ventilation (NPPV) and furosemide. A second exacerbation occurred on HCU Day 3, triggered by sympathetic activation and elevated blood pressure associated with sepsis, and improved with NPPV and fluid adjustment. A third exacerbation occurred on HCU Day 5 due to increased intravascular volume and sympathetic tone accompanying resolution of inflammation; although diuretics and respiratory support were administered, excessive diuresis led to hypovolemia, necessitating careful fluid management.

Wheezing was noted during respiratory deterioration, raising concern for possible chronic obstructive pulmonary disease exacerbation given his history of pulmonary emphysema and smoking history, and oral prednisolone was initiated. Respiratory status gradually improved, and oxygen therapy was discontinued by HCU Day 8.

By HCU Days 6 and 7, progressive leukopenia and anemia were observed during TAZ/PIPC therapy, raising suspicion of antibiotic-induced adverse effects. Therefore, the antimicrobial regimen was changed to sulbactam/ampicillin on HCU Day 8, after which hematologic abnormalities showed slight improvement without further progression. Recurrent heart failure exacerbations were observed in association with fluid shifts, accompanied by weight gain, lower extremity edema, jugular venous distention, and pleural effusion. Thoracentesis revealed serous fluid without evidence of infection.

The hepatic abscess gradually decreased in size with drainage, and the drainage characteristics improved. A follow-up CT performed two weeks after percutaneous drainage confirmed near-complete resolution of the hepatic abscess. Based on this favorable course and confirmation of fistula formation, the drainage catheter was removed by the gastroenterology team. Hypokalemia and hypophosphatemia were also noted and appropriately corrected. By HCU Day 16, vasopressors and NPPV were no longer required, and the patient was transferred out of the HCU with a favorable clinical course.

## Discussion

This case describes a patient with septic shock secondary to a liver abscess who developed recurrent exacerbations of heart failure on the background of HFpEF during treatment, followed by a relapse of shock after drainage. A notable feature of this case is that delayed fluid overload after initial resuscitation and post-source control hemodynamic deterioration emerged sequentially as major clinical challenges.

Adequate fluid resuscitation is recommended as the initial management of septic shock [4]. However, HFpEF is characterized by diastolic dysfunction and increased ventricular and vascular stiffness [5], which may reduce tolerance to volume loading and predispose patients to congestion with excessive fluid administration. In this case, although initial large-volume fluid resuscitation transiently improved hemodynamics, subsequent changes in vascular permeability and recovery processes may have led to intravascular refilling, whereby extravasated fluid returns to the intravascular space, thereby increasing circulating volume [6]. In addition to the initial fluid load, this mechanism may have contributed to delayed fluid overload and subsequent worsening of heart failure. Furthermore, following diuresis, a reduction in circulating blood volume led to recurrent hypotension requiring vasopressor escalation, highlighting the clinical challenge of determining the optimal timing for transition from resuscitation to de-resuscitation. In this case, fluid management was guided by an integrated assessment including inferior vena cava diameter, echocardiographic findings, body weight changes, urine output, and respiratory status. However, optimizing volume status remained difficult due to sepsis-related increases in vascular permeability and sympathetic activation. This clinical course illustrates the inherent tension in septic shock management between achieving adequate initial resuscitation and avoiding fluid overload. Therefore, in septic patients with HFpEF, fluid therapy after initial resuscitation should not be continued uniformly; instead, individualized fluid management based on integrated hemodynamic assessment and timely initiation of de-resuscitation is essential.

Source control is a cornerstone of sepsis management, and abscess drainage is a representative intervention [4,7]. However, invasive procedures may transiently exacerbate inflammatory responses and destabilize hemodynamics. In Gram-negative infections, pathogen-associated molecular patterns such as lipopolysaccharide can trigger host inflammatory responses, leading to vasodilation and circulatory failure [8]. In this case, the patient developed chills and hemodynamic deterioration after drainage, requiring re-escalation of vasopressors, suggesting that procedure-related augmentation of the inflammatory response contributed to the recurrence of circulatory failure. Additionally, this case suggests that a preexisting hepatic cyst may have become infected and progressed to an abscess, although alternative routes such as biliary or enteric bacterial translocation cannot be completely excluded. In this patient, no clear evidence of diverticulitis, active biliary obstruction, or other definitive intra-abdominal infectious sources was identified. This indicates that similar pathophysiological processes may occur in infections arising from cystic lesions. Even after successful source control, hemodynamic instability may transiently occur. Therefore, particularly in the periprocedural period, close monitoring and prompt hemodynamic interventions are essential with anticipation of possible shock recurrence.

## Conclusions

This case describes a patient with septic shock due to a liver abscess who developed recurrent exacerbations of heart failure on the background of preexisting heart failure, as well as relapse of shock following drainage. In the management of sepsis, patients with heart failure are prone to fluid overload; therefore, individualized fluid management is essential. Furthermore, even after source control, hemodynamic instability may occur transiently. Thus, careful periprocedural monitoring and appropriate hemodynamic management are crucial in clinical practice.
